# HIV-Infected Subjects With Poor CD4 T-Cell Recovery Despite Effective Therapy Express High Levels of OX40 and α4β7 on CD4 T-Cells Prior Therapy Initiation

**DOI:** 10.3389/fimmu.2018.01673

**Published:** 2018-07-18

**Authors:** Isaac Rosado-Sánchez, Inés Herrero-Fernández, Miguel Genebat, Jorge Del Romero, Melchor Riera, Daniel Podzamczer, Julián Olalla, Francesc Vidal, Mª Angeles Muñoz-Fernández, Manuel Leal, Yolanda M. Pacheco

**Affiliations:** ^1^Institute of Biomedicine of Seville, IBiS, Virgen del Rocío University Hospital/CSIC/University of Seville, Seville, Spain; ^2^Centro de Salud Sandoval, Madrid, Spain; ^3^Son Espases University Hospital, Palma de Mallorca, Spain; ^4^Bellvitge University Hospital, L’Hospitalet de Llobregat, Spain; ^5^Costa del Sol Hospital, Marbella, Spain; ^6^Joan XXIII University Hospital, IISPV, Rovira I Virgili University, Tarragona, Spain; ^7^Section Immunology, Laboratorio InmunoBiología Molecular, Hospital General Universitario Gregorio Marañón, Madrid, Spain; ^8^Spanish HIV HGM BioBank, Madrid, Spain; ^9^Instituto de Investigación Sanitaria Gregorio Marañón (IiSGM), Madrid, Spain; ^10^CIBER-BBN, Madrid, Spain; ^11^Internal Medicine Service, Viamed-Santa Ángela Hospital, Seville, Spain

**Keywords:** immunodiscordant response to combined antiretroviral treatment, low CD4 recovery, homeostatic parameters, homeostatic proliferation, OX40, α4β7, CD4 T-cell homeostasis, HIV

## Abstract

**Background:**

HIV-infected subjects with suboptimal CD4 restoration despite suppressive combined antiretroviral treatment (cART) (immunodiscordant subjects) have been classically characterized after a variable period of time under cART. Recently, we have reported that an increased frequency of proliferating CD4 T-cells in these subjects is already present before the cART onset. The potential contribution of peripheral compensatory homeostatic proliferation (HP) is yet unknown. We aimed to analyze the expression of HP-related cellular markers on CD4 T-cells of immunodiscordant subjects before cART.

**Methods:**

We analyzed the expression of OX40 and α4β7 on peripheral CD4 T-cells from immunodiscordant and control subjects (*n* = 21 each group) before cART initiation, and also on available follow-up samples (after 24 month of suppressive cART). Additionally, we tested the expression of these markers in an *in vitro* system for the study of human HP processes.

**Results:**

Immunodiscordant subjects showed increased levels of OX40 and α4β7 on CD4 T-cells before cART initiation. While the cART tended to reduce these levels, immunodiscordant subjects still maintained comparatively higher levels of OX40 and α4β7 after 24 months under suppressive cART. These HP-related markers were upregulated *in vitro* during the human HP, especially during the fast HP.

**Conclusion:**

Our results are compatible with exacerbated HP processes in immunodiscordant subjects, already before the cART onset.

## Introduction

Those subjects starting combined antiretroviral treatment (cART) with low CD4 counts and maintaining such low levels despite persistent suppression of viremia (immunodiscordant subjects with low-level CD4) constitute a particularly relevant population among HIV-infected subjects. They show increased rates of clinical complications than those subjects having a subsequent proper CD4-cell recovery (1, 2). Unfortunately, the mechanisms triggering such response still remain unknown.

These immunodiscordant subjects have been exhaustively studied after cART onset, when immunodiscordant response to cART has already taken place (3, 4), but less is known about immune alterations preexisting before cART initiation and, therefore, preceding such anomalous response to the treatment. Our group has been a pioneer in reporting immune alterations preceding the low CD4 recovery in this scenario (5, 6). Interestingly, these immunodiscordant subjects showed increased frequencies of proliferating CD4 T-cells already before cART initiation (5), denoting an early impairment of CD4 T-cell homeostasis, which deserves further research.

Globally, in T-cell lymphopenia conditions, a peripheral compensatory homeostatic proliferation (HP) of preexisting T-cells occurs (7). Two different types of HP have been extensively described: a commensal/self-antigen-driven fast HP and a cytokine-driven slow HP (mainly IL7 and IL15) (8), although both types of stimuli can synergize impacting each type of HP (9, 10). Recent works have found that fast HP specifically upregulates the tumor necrosis factor receptor superfamily member 4 (OX40) (11, 12), and that the blockade of OX40–OX40L interaction hamper the fast HP, taking place mainly in mesenteric lymph nodes (11). In this sense, a gut-homing imprinting (α4β7-integrins) has also been associated with the fast HP (11), but also with the slow HP in HIV-infected subjects after IL7 administration (13).

Our aim was to analyze the expression of OX40 and α4β7 on CD4 T-cells of immunodiscordant subjects before the cART onset. Additionally, in an *in vitro* model for the study the human HP we explored the modulation of these markers during such processes.

## Materials and Methods

### Subjects and Samples

Samples were selected from the Spanish AIDS Research Network Cohort (CoRIS) (14) and kindly provided by its HIV BioBank (15). Samples were processed following current procedures and frozen immediately after their reception. All patients participating in the study gave their informed consent and institutional ethical committees approved protocols. We selected pre-cART samples (up to 6 months before cART onset) of peripheral blood mononuclear cells from antiretroviral-naïve subjects who had started cART with <200 CD4/mm^3^ and did not reach 250 CD4/mm^3^ after 24 months of suppressive treatment (Low CD4 recovery subjects; LR-subjects), and subjects who also started cART with <200 CD4/mm^3^ but achieved more than 250 CD4/mm^3^ after 24-months of suppressive treatment (High CD4 recovery subjects, HR-subjects). Both groups of subjects were matched by sex, age, viral load, and baseline CD4 counts. Finally, 21 subjects per group were included (a flow chart of subject’s selection is shown in Figure S1 in Supplementary Material). Depending on sample availability, additional post-cART follow-up samples (24 ± 6 months on cART) were also analyzed.

For the *in vitro* T-cell culture experiments, buffy coats from HIV-1 and Hepatitis C Virus (HCV) seronegative anonymous donors from the *Centro Regional de Transfusión Sanguínea de Sevilla-Huelva y Banco de Tejidos* (Seville, Spain) were obtained. The study was approved by the local Ethical Committee and was performed according to the European Union guidelines and the Declaration of Helsinki.

### Immunophenotyping

After thawing, cells were stained with surface antibodies (detailed in Supplemental Methods in Supplementary Material). Viable cells were identified using LIVE/DEAD fixable Aqua Blue Dead Cell Stain (Life Technologies, USA). The homing-related molecules α4-integrin and β7-integrin, and the TNFR-related molecule OX40 (CD134) were analyzed. A schematic diagram of the gating strategy used is shown in Figure S2 in Supplementary Material. Flow cytometry was performed on a LSR Fortessa (BD, USA). The analysis was performed using FlowJo version 9.2 (Tree Star) and data are expressed as frequencies (%).

### *In Vitro* Culture of Human Lymphocytes

The *in vitro* culture used for the study of HP was performed as previously described (10). Briefly, untouched CFSE-dyed naïve CD4 T-cells were seeded in supplemented medium (RPMI 1640 supplemented with 10% fetal bovine serum, 1.7 mM glutamine, 100 µl/ml streptomicine and 100 U/ml peniciline), and stimulated with irradiated autologous aAPCs (1/1 ratio aAPCs/Naïve) and rIL7 (10 ng/ml). Finally, after 10 days of culture, cells were collected and immunophenotyped.

### Statistical Analyses

Variables from *in vivo* analyses were expressed as median values and interquartile range. Mann–Whitney *U* and Wilcoxon rank test were used to analyze unpaired and paired comparisons, respectively. For analyses of cellular markers in the culture, the variables were expressed as mean and SD. A *p-*value < 0.05 was considered statistically significant. Statistical analysis was performed using the Statistical Package for the Social Sciences software (SPSS 22.0, USA). Graphs were created using Prism version 5.0 (GraphPad Software, USA).

## Results

### Higher Levels of OX40 and α4β7 on CD4 T-Cells of LR-Subjects Before and After cART Instauration

**Figure 1:**
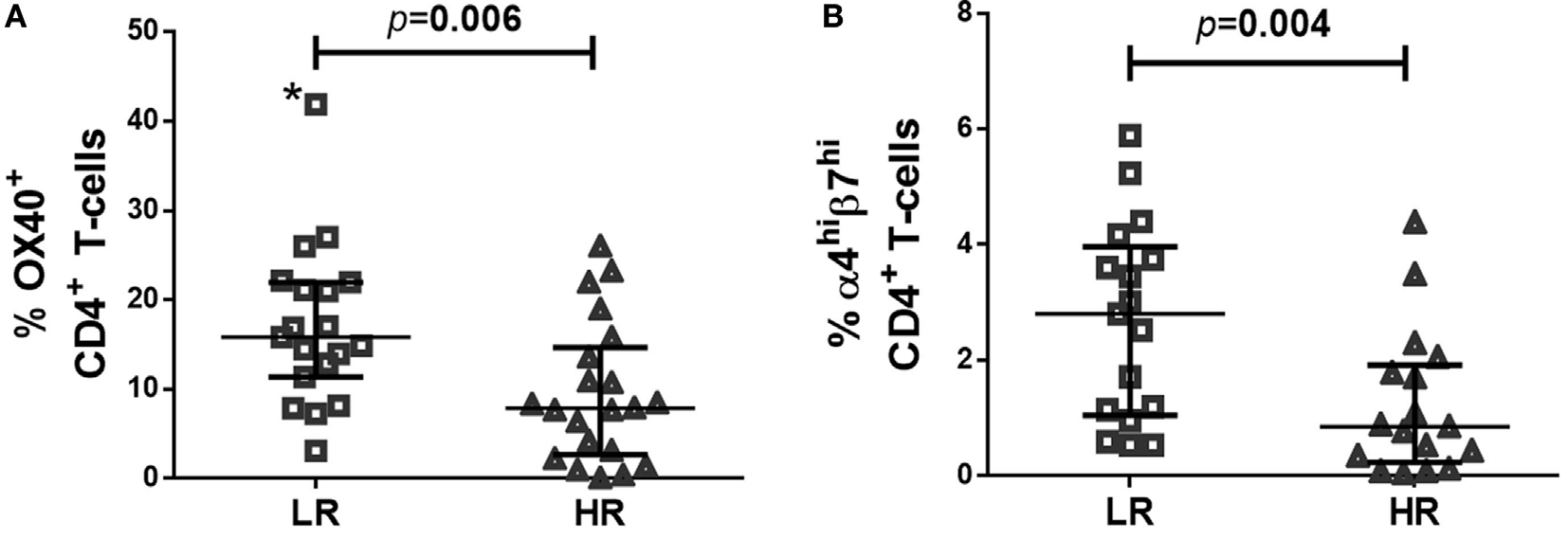
Expression of OX40 and α4β7 in CD4 T-cells of LR-subjects at combined antiretroviral treatment onset. **(A)** Frequency of OX40^+^CD4^+^ T-cells; **(B)** Frequency of α4^hi^β7^hi^ CD4^+^ T-cells. After excluding outlier value (*) statistical significance was *p* = 0.009. Data were determined using multiparametric flow cytometry and are expressed as median value and interquartile range (IQR). Mann–Whitney test was used for the statistical significance calculation.

The baseline clinical characteristics of LR- and HR-subjects are described elsewhere (5). Most importantly, groups did not differ statistically in matched variables, nor did in other clinical variables as time from HIV diagnosis or route of transmission. Before cART initiation, LR-subjects showed higher frequencies of OX40^+^ CD4^+^ T-cells (Figure 1A), and a trend to higher frequencies of α4^+^β7^+^ CD4^+^ T-cells (LR = 9.2 [5.2–12.1]; HR = 5.0 [2.5–9.0]; *p* = 0.051), that reached statistical significance when comparing frequencies of α4^hi^β7^hi^ CD4^+^ T-cells (Figure 1B).

According to the classification criteria, after 24 months under cART, LR-, and HR-subjects had achieved 208 [141–234] and 429 [372–524] CD4 T-cells/mm^3^, respectively. Additionally, we longitudinally compared samples of 10 LR and 8 HR-subjects. Suppressive cART tended to reduce levels of OX40 (*p* = 0.069 and *p* = 0.036 for longitudinal comparisons of LR- and HR-subjects, respectively) and α4^hi^β7^hi^ (*p* = 0.093 and *p* = 0.116 for longitudinal comparisons of LR- and HR-subjects, respectively). However, LR-subjects still sustained higher levels than HR-subjects for both markers, OX40 (LR = 10.0 [11.3–7.1]; HR = 5.8 [7.4–5.0]; *p* = 0.034) and α4^hi^β7^hi^ (LR = 4.9 [6.4–2.7]; HR = 1.5 [3.7–1.0]; *p* = 0.025).

### OX40 and α4β7-Integrins Are Upregulated During HP of Human CD4 T-Cells

**Figure 2:**
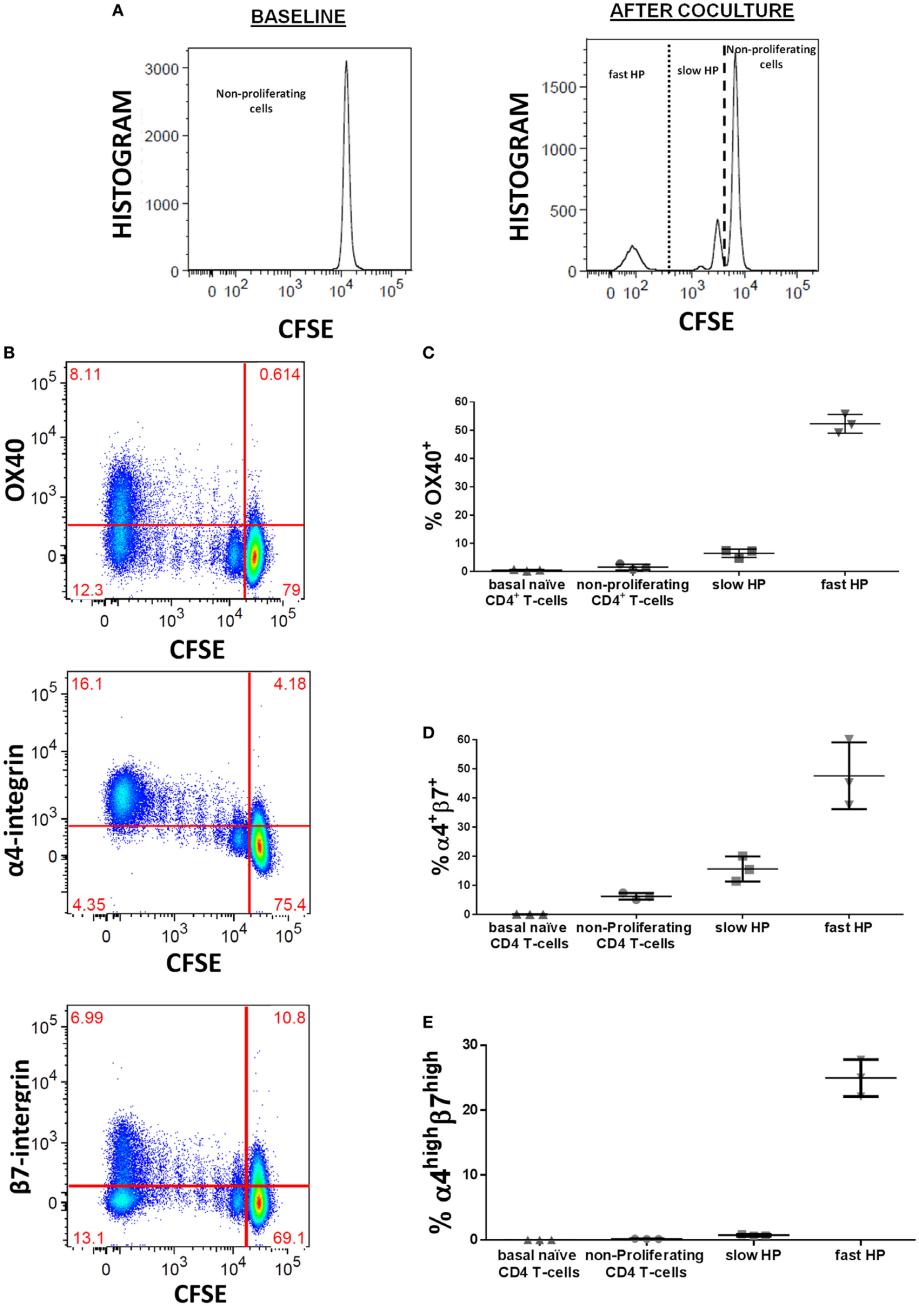
OX40 and α4β7 expression during the homeostatic proliferation (HP) process of human naïve CD4 T-cells. Untouched CFSE-stained naïve CD4 T-cells were culture in presence of rIL7 (10 ng/ml) and with irradiated autologous APCs (1/1 ratio): **(A)** representative CFSE histograms used for identification of different types of HP at baseline (0 day) and after 10 days of culture, **(B)** OX40, α4-integrin, and β7-integrin upregulation during HP, **(C)** frequencies of OX40 in the different types of HP subsets, **(D)** frequencies of α4^+^β7^+^ in the different types of HP subsets, **(E)** frequencies of α4^high^β7^high^ in the different types of HP subsets. Frequencies of both OX40 and α4β7 were calculated after background substation from baseline condition. Data are expressed as mean value and SD.

We explored the modulation of these HP-related markers previously associated with the HP using an *in vitro* cell culture specifically designed to study such processes in human T-lymphocytes (10). This culture system allowed us analyzing the expression of such markers as a consequence of each type of HP, the slow and the fast HP. Dilution of CFSE was used for identification of different types of HP (Figure 2A). Especifically, fast HP was defined as viable CD4 T-cells which fully diluted CFSE (and acquired memory phenotype), while slow HP was defined as viable CD4 T-cells which partially diluted CFSE (but maintained naïve phenotype) (10). We observed that OX40, α4-integrin, and β7-integrin were upregulated on human lymphocytes during the HP induced by homeostatic stimuli (Figure 2B). OX40 was mainly expressed in cells undergoing fast HP (Figure 2C). Similarly, when considering the coexpression of α4 and β7 as a single marker, a greater specificity in discriminating fast and slow HP was observed for α4^high^β7^high^ compared with α4^+^β7^+^ (Figures 2D,E). As it can be observed, the percentages of OX40^+^ and α4^high^β7^high^ cells were around the 50 and 30%, respectively, among those generated during the fast HP, whereas this percentage was lower than 10 and 2%, respectively, among those generated by slow HP.

## Discussion

We report here that HIV-infected subjects with low CD4 T-cell counts and immunodiscordant response to cART present higher expression of OX40 and α4β7 on CD4 T-cells before and after suppressive cART than their control group. These markers, which have been related to HP mainly in animal models, were also upregulated during HP of human T-lymphocytes, mostly during fast HP, similarly as it occurs *in vivo*.

While a higher expression of markers of activation, senescence, exhaustion, and proliferation on CD4 T-cells has been extensively reported in immunodiscordant subjects after cART (16–19), they only differed in a higher expression of Ki67 on CD4 T-cells when all these markers were compared before the cART onset (5). Interestingly, in this comparative study, microbial translocation, HCV coinfection, baseline viral load, or viral tropism did not differ either between groups (5). Since HP is an additional potential cause of a higher proliferative status, especially in lymphopenia scenarios (7) such as HIV-infection (20), we aimed here to explore the expression of HP-related markers in this particular context.

It is well-known that immunodiscordant subjects present higher frequencies of Treg (21, 22) and Th17 (23, 24) after cART initiation. Additionally, we have recently reported that increased frequencies of both Treg and Th17 are already present before cART initiation (5, 6) in these subjects. Interestingly, the fast HP is able to generate Treg (10, 25) and Th17 (11), and the costimulatory molecule OX40 is a key mediator in such process (11, 12). Moreover, we report here that OX40 is also upregulated specifically during the human fast HP. Thus, the higher frequency of OX40 + CD4 T-cells found now in immunodiscordants, together with the previous data, point to an exacerbation of the fast HP process in this scenario. Importantly, the OX40–OX40L axis has been associated with the production of proinflammatory cytokines such as IL6 by dendritic cells (26) and with inflammatory disorders (27), suggesting that HP, through OX40 upregulation, could also contribute to the higher inflammation observed in this scenario (5). Also of relevance, inflammatory cytokines such as IL6 have been reported to increase proliferation of memory CD4 T-cells but also lead to downregulation of CD127 (IL7 receptor-α) (28). Therefore, inflammatory cytokines induced by the OX40–OX40L interaction could theoretically exacerbate fast HP and likewise hamper slow HP. However, further studies are necessary to a better understanding of the specific relationship between inflammatory cytokines and HP.

The T-cell trafficking from blood to the gut-associated lymphoid tissue (GALT) is mediated by the β7- and α4-integrins among other homing-molecules (29, 30). Here, we have found a higher frequency of α4^high^β7^high^ CD4 T-cells in immunodiscordant subjects before and after suppressive cART. Using an *in vitro* system, we previously reported that both, fast and slow human HP upregulate β7-integrin (10). Additionally, we have now observed that human HP also upregulates α4-integrin and that α4^+^β7^+^ CD4 T-cells, but especially α4^high^β7^high^ CD4 T-cells, are mainly generated during fast HP. Also relevant, immunodiscordant subjects before cART initiation did not show higher levels of peripheral IL7 (5), the main cytokine driving slow HP. Altogether is consistent with the fast HP as the more plausible HP mechanism leading to the increased expression of α4β7 on CD4 T-cells in this particular context. Interestingly, α4β7-integrins favor the HIV colonization of GALT and the establishment of mucosal HIV reservoir (31). In this line, the higher expression of α4β7 in CD4 T-cells recently reported in a different cohort of immunodiscordant subjects under suppressive cART, was correlated with a higher proviral HIV load (24). Additionally, the expression of OX40 on CD4 T-cells has been associated with a high metabolic activity which is ultimately associated with a higher permissibility to HIV infection (32), and HP process itself has been also associated with higher amounts of HIV provirus (33). Therefore, all these data suggest that the higher HP before of cART onset in immunodiscordant subjects could imply a higher proviral HIV load, but additional studies are necessary to clarify this topic.

Our cohort study has a limited number of subjects, but it is counterbalanced by a clean case:control design allowing the adjustment by the main associated risk factors to the immunodiscordant response to cART. On the other hand, an *in vivo* human model for the study of HP, including anatomical support, would have been preferable but is not easily affordable. However, the *in vitro* model was useful to corroborate the upregulation of studied markers during human HP.

In summary, we have found that immunodiscordant subjects with low-level CD4 show an early and specific increase in the expression of OX40 and α4β7 on CD4 T-cells. The longitudinal exploration allowed proving that such alteration persists after suppressive cART, despite some extent of improvement. These novel features and our previous related data are all consistent with a promptly enhanced HP in this scenario.

## Ethics Statement

The name of the ethics committee that approved the study is “CEI de los hospitales universitarios Vírgen Macarena-Virgen del Rocío.” All studied subjects gave a written informed consent before donate their samples. The study was approved by the local Ethical Committee and was performed according to the European Union guidelines and the Declaration of Helsinki.

## Author Contributions

IR-S contributed to the design, performed research, analyzed, and interpreted data. IR-S and YP wrote the manuscript. IH-F and MG contributed to several analyses and/or experiments. JR, MR, DP, and JO represent contribution to sample and data collection by RIS BB/CoRIS. MM-F and FV critically reviewed the manuscript. ML and YP designed the study and contributed to data interpretation. YP conceived the study.

## Conflict of Interest Statement

Part of the methods used in this article, specifically the culture system, is pending to be patented. However, the markers studied in this work are not included in such patent. The authors declare that the research was conducted in the absence of any commercial or financial relationships that could be construed as a potential conflict of interest.
