# Supplementary material for: HIV-Infected Subjects With Poor CD4 T-Cell Recovery Despite Effective Therapy Express High Levels of OX40 and α4β7 on CD4 T-Cells Prior Therapy Initiation
Source: Front Immunol. 2018 Jul 18;9:1673. doi: 10.3389/fimmu.2018.01673 (PMC6058017; doi:10.3389/fimmu.2018.01673)
Supplement: Supplementary file 1 [file Data_Sheet_1.doc]

**SUPPLEMENTAL DATA**

**Supplemental Methods**

Multiparametric Flow Cytometry materials

Extracellular and intracellular antibodies used in PBMCs immunophenotyping were:
anti-CD3 (Clone: SK7), anti-CD4 (Clone: L200), anti-β7-integrin (Clone: FIB504), (BD bioscience, USA), anti-OX40 (Clone: Ber-ACT35), anti-CD49d/α4-integrin (Clone: 9F10) (Biolegend, USA). Isotype controls for α4-integrin, β7-integrin and OX40 were included in each experiment.

Extracellular antibodies used in cellular culture experiments were: anti-CD3 (Clone: SK7), anti-CD45RA (Clone: HI100), anti-β7-integrin (Clone: FIB504) (BD bioscience, USA), anti-OX40 (Clone: Ber-ACT35), anti-CD49d/α4-integrin (Clone: 9F10) (Biolegend, USA). Isotype controls for OX40, β7-integrin and α4-integrin were used when necessary.

**Supplemental Figures**

**
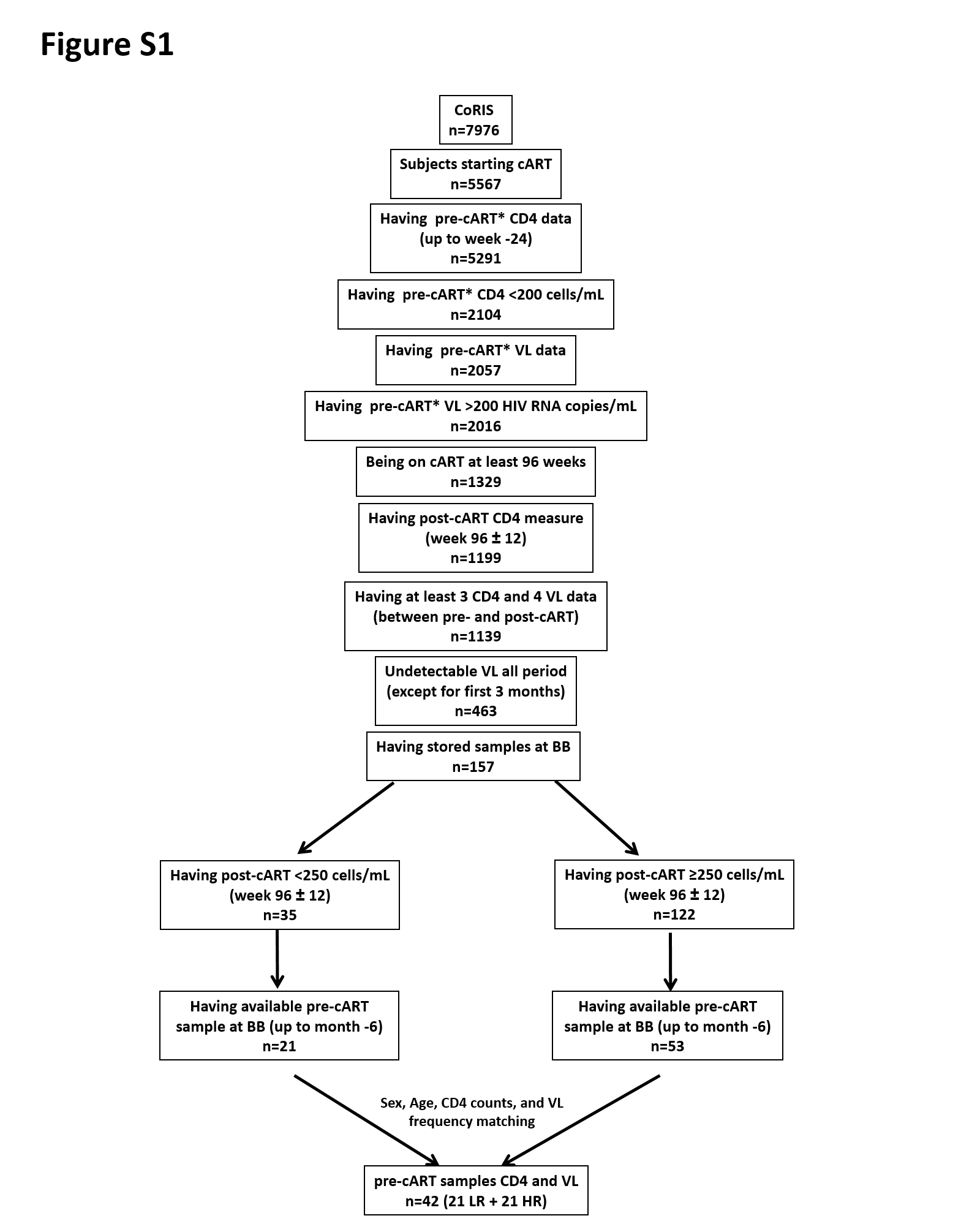
**

**Figure S1:** Flow-chart selection of pre-cART samples of LR- and HR-subjects from CoRIS-RIS (updated on October 31, 2011) and Spanish BioBank.


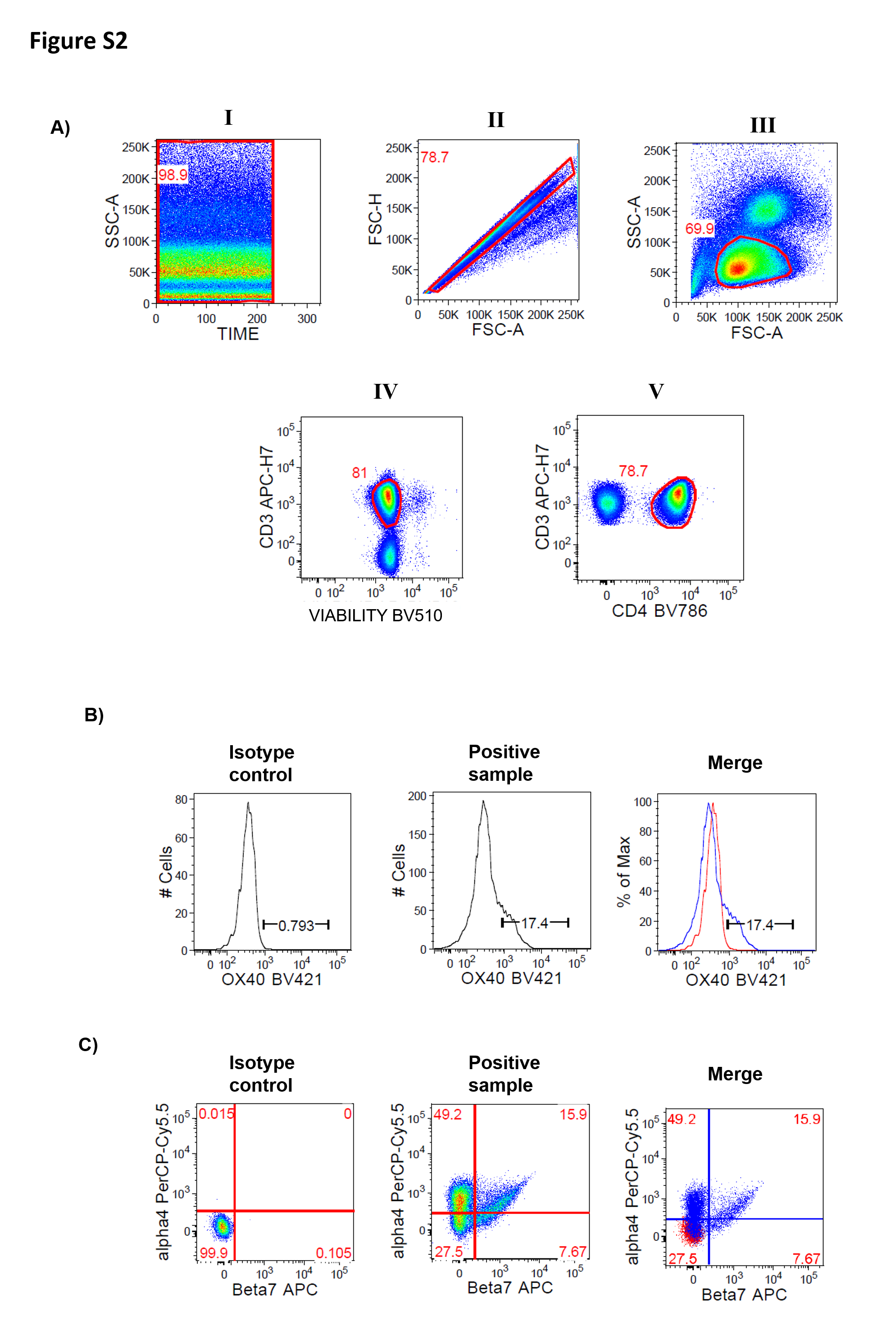


**Figure S2:** Flow cytometry gating strategies. Representative examples of the gating strategy for: **A)** CD4 T-cells; **B)** OX40+ CD4 T-cells; **C)** α4^+^β7^+^ CD4 T-cells.

**Annex: Centers and investigators involved in CoRIS:**

**Executive committee**: Santiago Moreno, Julia del Amo, David Dalmau, Maria Luisa Navarro, Maria Isabel González, Jose Luis Blanco, Federico Garcia, Rafael Rubio, Jose Antonio Iribarren, Félix Gutiérrez, Francesc Vidal, Juan Berenguer, Juan González.

**Fieldwork, data management and analysis:** Paz Sobrino, Victoria Hernando, Belén Alejos, Débora Álvarez, Inma Jarrín, Yaiza Rivero, Cristina González.

**Spanish BioBanK HIV (**<http://hivhgmbiobank.com/>**):** M Ángeles Muñoz-Fernández, Isabel García-Merino, Coral Gómez Rico, Jorge Gallego de la Fuente y Jose Luis Jiménez.

**Participating centres:**

**Hospital General Universitario de Alicante (Alicante):** Joaquín Portilla, Esperanza Merino, Sergio Reus, Vicente Boix, Livia Giner, Carmen Gadea, Irene Portilla, Maria Pampliega, Marcos Díez, Juan Carlos Rodríguez, Jose Sánchez-Payá.

**Hospital Universitario de Canarias (San Cristobal de la Laguna):** Juan Luis Gómez, Jehovana Hernández, María Remedios Alemán, María del Mar Alonso, María Inmaculada Hernández, Felicitas Díaz-Flores, Dácil García, Ricardo Pelazas. , Ana López Lirola.

**Hospital Universitario Central de Asturias (Oviedo):** Victor Asensi, Eulalia Valle, José Antonio Cartón., Maria Eugenia Rivas Carmenado.

**Hospital Universitario 12 de Octubre (Madrid):** Rafael Rubio, Federico Pulido, Otilia Bisbal, Asunción Hernando, Maria Lagarde, Mariano Matarranz, Lourdes Dominguez, Laura Bermejo, Mireia Santacreu.

**Hospital Universitario de Donostia (Donostia-San Sebastián):** José Antonio Iribarren, Julio Arrizabalaga, María José Aramburu, Xabier Camino, Francisco Rodríguez-Arrondo, Miguel Ángel von Wichmann, Lidia Pascual Tomé, Miguel Ángel Goenaga, Mª Jesús Bustinduy, Harkaitz Azkune Galparsoro. , Maialen Ibarguren, Maitane Umerez.

**Hospital General Universitario De Elche (Elche):** Félix Gutiérrez, Mar Masiá, Sergio Padilla, Andrés Navarro, Fernando Montolio, Catalina Robledano, Joan Gregori Colomé, Araceli Adsuar, Rafael Pascual, Marta Fernández, Elena García., Jose Alberto García, Xavier Barber.

**Hospital General Universitario Gregorio Marañón (Madrid):** Juan Berenguer, Juan Carlos López Bernaldo de Quirós, Pilar Miralles, Isabel Gutiérrez, Margarita Ramírez, Belén Padilla, Paloma Gijón, Ana Carrero, Teresa Aldamiz-Echevarría, Francisco Tejerina, Francisco Jose Parras, Pascual Balsalobre, Cristina Diez.

**Hospital Universitari de Tarragona Joan XXIII (Tarragona):** Francesc Vidal, Joaquín Peraire, Consuelo Viladés, Sergio Veloso, Montserrat Vargas, Miguel López-Dupla, Montserrat Olona, Anna Rull, Esther Rodriguez-Gallego, Verónica Alba.

**Hospital Universitario y Politécnico de La Fe (Valencia):** Marta Montero Alonso, José López Aldeguer, Marino Blanes Juliá, Mariona Tasias Pitarch, Iván Castro Hernández, Eva Calabuig Muñoz, Sandra Cuéllar Tovar, Miguel Salavert Lletí, Juan Fernández Navarro.

**Hospital Universitario La Paz (Madrid):** Juan González, Ignacio Bernardino de la Serna, José Ramón Arribas, María Luisa Montes, Jose Mª Peña, Blanca Arribas, Juan Miguel Castro, Fco Javier Zamora, Ignacio Pérez, Miriam Estébanez, Silvia García, Marta Díaz, Natalia Stella Alcáriz, Jesús Mingorance, Dolores Montero, Alicia González, Maria Isabel de José.

**Hospital Universitari MutuaTerrassa (Terrasa):** David Dalmau, Angels Jaén, Montse Sanmartí, Mireia Cairó, Javier Martinez-Lacasa, Pablo Velli, Roser Font, Mariona Xercavins, Noemí Alonso.

**Hospital Universitario de La Princesa (Madrid):** Ignacio de los Santos, Jesús Sanz Sanz, Ana Salas Aparicio, Cristina Sarriá Cepeda, Lucio Garcia-Fraile Fraile.

**Hospital Universitario Ramón y Cajal (Madrid):** Santiago Moreno, José Luis Casado, Fernando Dronda, Ana Moreno, María Jesús Pérez Elías, Cristina Gómez Ayerbe, Carolina Gutiérrez, Nadia Madrid, Santos del Campo Terrón, Paloma Martí, Uxua Ansa, Sergio Serrrano, Maria Jesús Vivancos.

**Hospital General Universitario Reina Sofía (Murcia):** Alfredo Cano, Enrique Bernal, Ángeles Muñoz.

**Hospital Nuevo San Cecilio (Granada):** Federico García, José Hernández, Alejandro Peña, Leopoldo Muñoz, Ana Belén Pérez, Marta Alvarez, Natalia Chueca, David Vinuesa, Jose Angel Fernández.

**Centro Sanitario Sandoval (Madrid):** Jorge Del Romero, Carmen Rodríguez, Teresa Puerta, Juan Carlos Carrió, Mar Vera, Juan Ballesteros.

**Hospital Universitario Son Espases (Palma de Mallorca):** Melchor Riera, Maria Peñaranda, Maria Leyes, Mª Angels Ribas, Antoni A Campins, Carmen Vidal, Francisco Fanjul, Javier Murillas, Francisco Homar.

**Hospital Universitario Virgen de la Victoria (Málaga):** Jesús Santos, Manuel Márquez, Isabel Viciana, Rosario Palacios, Isabel Pérez, Carmen Maria González.

**Hospital Universitario Virgen del Rocío (Sevilla):** Pompeyo Viciana, Nuria Espinosa, Luis Fernando López-Cortés.

**Hospital Universitario de Bellvitge (Hospitalet de Llobregat):** Daniel Podzamczer, Elena Ferrer, Arkaitz Imaz, Juan Tiraboschi, Ana Silva, Maria Saumoy.

**Hospital Costa del Sol (Marbella):** Julián Olalla, Alfonso del Arco, Javier de la torre, José Luis Prada, José María García de Lomas Guerrero.

**Hospital General Universitario Santa Lucía (Cartagena):** Onofre Juan Martínez, Francisco Jesús Vera, Lorena Martínez, Josefina García, Begoña Alcaraz, Amaya Jimeno.

**Complejo Hospitalario Universitario a Coruña (Chuac) (A Coruña):** Eva Poveda, Berta Pernas, Álvaro Mena, Marta Grandal, Ángeles Castro, José D. Pedreira.

**Hospital Universitario Virgen de la Arrixaca (El Palmar):** Carlos Galera, Helena Albendin, Aurora Pérez, Asunción Iborra, Antonio Moreno, Maria Ángeles Campillo, Asunción Vidal.

**Hospital Universitario Infanta Sofia (San Sebastian de los Reyes):** Inés Suárez-García, Eduardo Malmierca.

**Complejo Hospitalario de Jaén (Jaen):** Mohamed Omar Mohamed-Balghata, Maria Amparo Gómez Vidal.
